# Supplementary figures and images for: Covid-19 and excess mortality in medicare beneficiaries
Source: PLoS One. 2022 Feb 2;17(2):e0262264. doi: 10.1371/journal.pone.0262264 (PMC8809573; doi:10.1371/journal.pone.0262264)

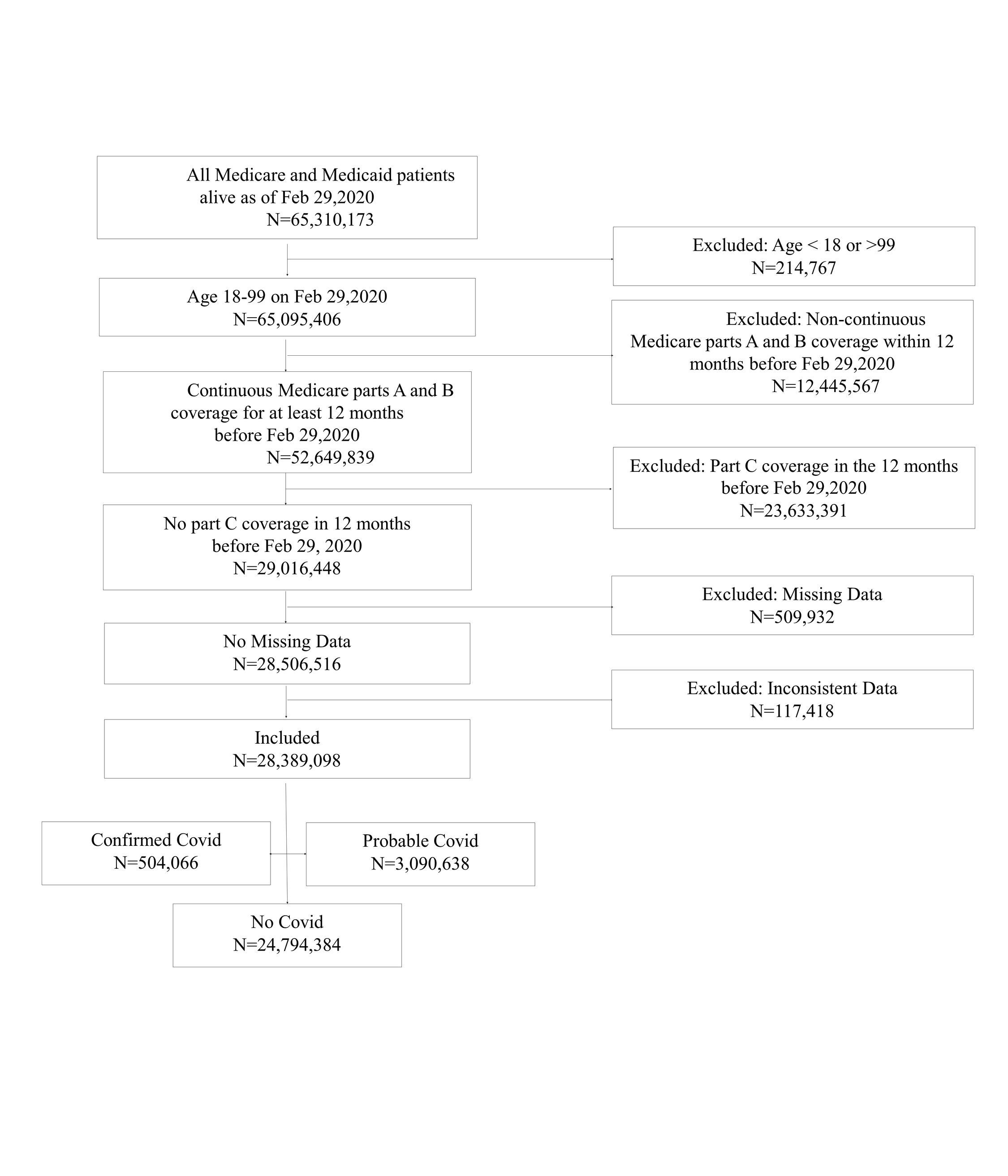

Supplement: S1 Fig — Confirmed Covid-19 cases were identified consistent with CMS guidance using ICD-10-CM codes for Covid-19 (B97.29 before April 1, 2020 and U07.1 thereafter) as a primary or secondary diagnosis between March 1, 2020 and September 30, 2020 [22]. Probable Covid-19 infection cases were identified using ICD-10-CM codes consistent with the CDC guidance (Z20.828) and WHO recommendations (U07.2) [23, 24]. Subjects were excluded for missing data if values for any baseline characteristic used in the study were missing (i.e., age, sex, ethnicity, location of care, zip code derived measures, dates of coverage, or baseline risk of 9 month mortality assessed with the Risk Stratification Index (RSI).) Additionally, we excluded subjects whose records had inconsistent values among source files containing similar variables such as birth date and sex. (TIF) [file pone.0262264.s001.tif]

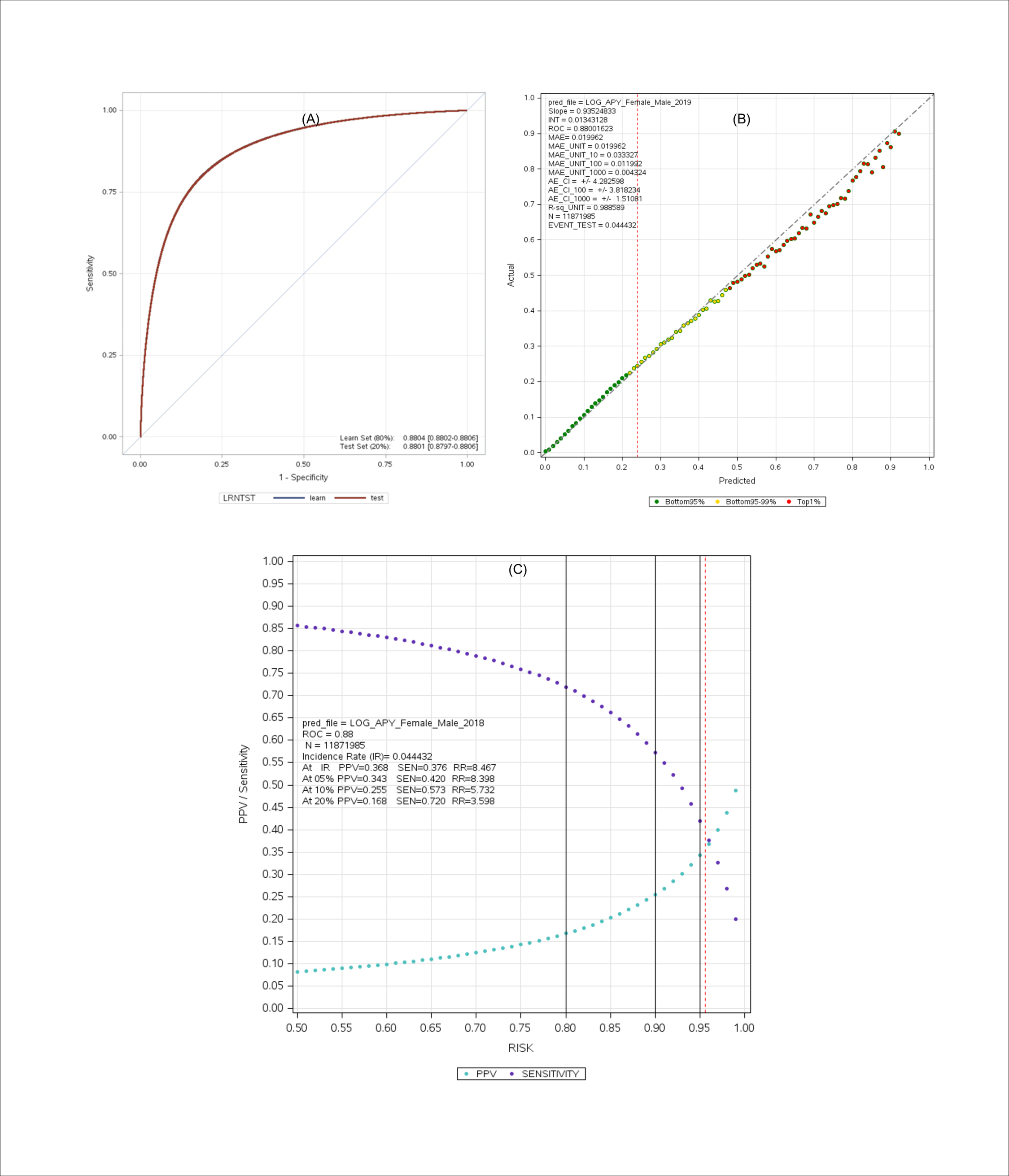

Supplement: S2 Fig — Panel A: ROC curve, Panel B Calibration plot, Panel C Sensitivity and Positive Predicted Value vs probability of mortality. (A) Area Under the Receiver Operating Curve (AuROC) for the development Learn Set (80% of 2018 Set) was 0.88 (95% Confidence Interval of [0.88–0.88]). AuROC for the prospective Test Set (20% of 2018 Set) was 0.88 (95% Confidence Interval of [0.88–0.88]. Similar performance in the Test Set compared to the Learn Set supports a lack of overfitting in the development of the predictor. (B) The calibration plot displays the mean actual vs predicted 1 year mortality for populations clustered in increments of 1% probability of mortality. Dark green, light green, and red dots are populations of the lowest 95%, 95%-99%, and top 1% risk of mortality. The diagonal line identifies the domain of ideal performance where actual and expected mortality rates are equal for a population. The performance of this index is very close to ideal performance for approximately 99% of the population. Tabulated metrics: The sample size in this test set (N) was 11,871,985 with an incidence of 1yr mortality (Event_Test) of 4.4%. The Slope and Intercept (INT) fit of the data are 0.94 and 0.01, respectively. The area under the Receiver Operating Curve was 0.88. The Mean Average Error (MEA) from cluster coordinates (i.e., (expected, actual) couplets) to the identity line was calculated for the database divided into populations grouped from the riskiest to least risky subjects using cluster sizes ranging from 1 (i.e., each individual as a cluster) to 1000 neighboring subjects (e.g., MAE to MAE_1000). The 95% Confidence Interval (CI) for the fits of these populations to the identify line is tabulated (i.e., AE_CI to AE_CI_1000). Rsq_unit is a goodness of fit measure of individual results to the ideal line. (C) Positive Predictive Accuracy (blue dots) and Sensitivity (purple dots) versus the fraction of population, sorted by the risk of 1 year mortality. The vertical red line indic [file pone.0262264.s002.tif]

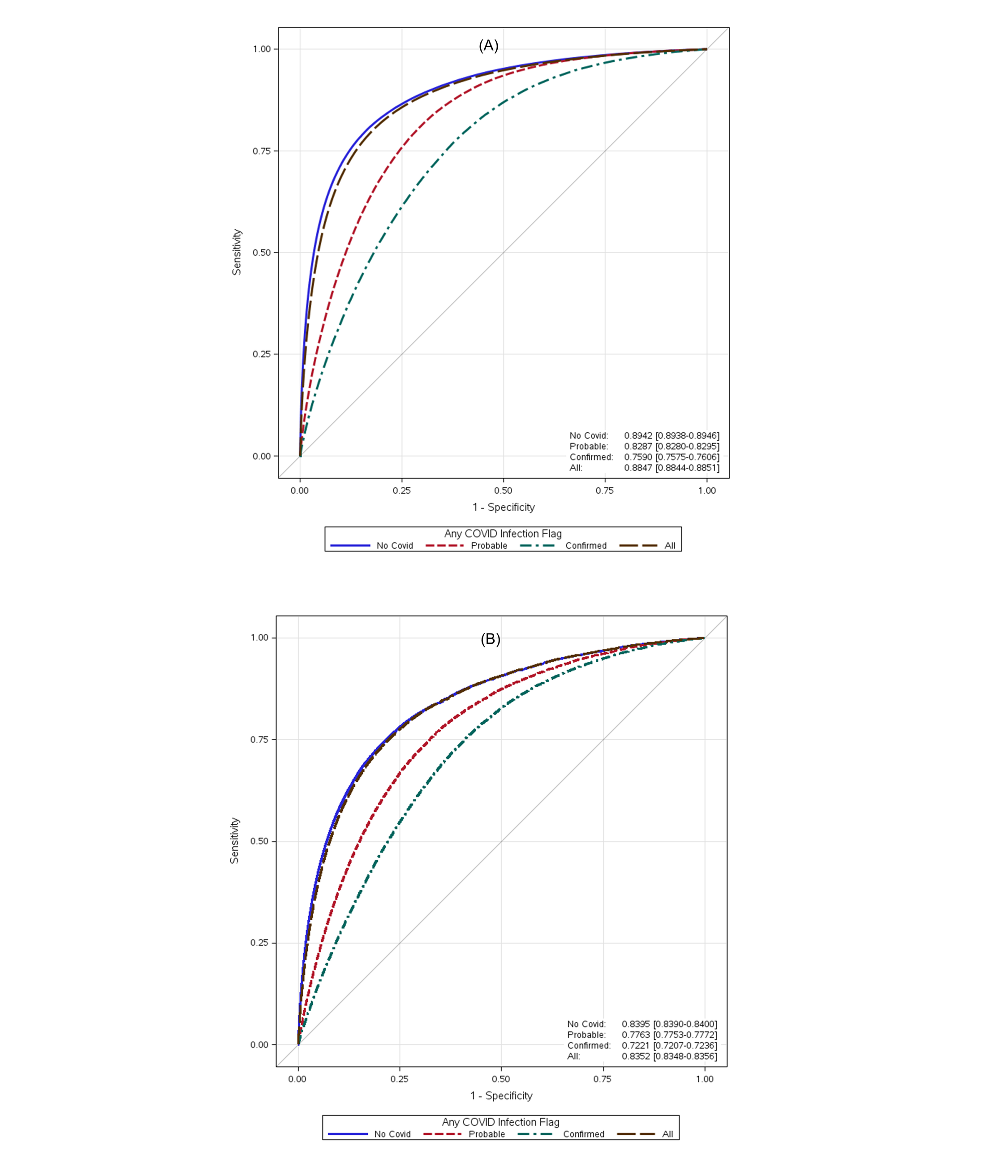

Supplement: S3 Fig — Comparison of RSI (Panel A) and Chronic Condition based models (Panel B) on 2020 population. ROC curves for all subjects, No Covid-19, Probable Covid-19 and Confirmed Covid-19 populations. Confirmed Covid-19 cases were identified consistent with CMS guidance using ICD-10-CM codes for Covid-19 (B97.29 before April 1, 2020 and U07.1 thereafter) as a primary or secondary diagnosis between March 1, 2020 and September 30, 2020 [22]. Probable Covid-19 infection cases were identified using ICD-10-CM codes consistent with the CDC guidance (Z20.828) and WHO recommendations (U07.2) [23, 24]. (A,B) ROCs display the sensitivity vs. 1 –specificity in detecting patients who died within 9 months after prediction from February 29,2020 (baseline). The areas under each ROC, with their corresponding 95% confidence intervals, are tabulated in the lower right of each figure. Predictions using RSI yielded better performance (A) than those using a model based on age, sex and chronic conditions (B). (TIF) [file pone.0262264.s003.tif]

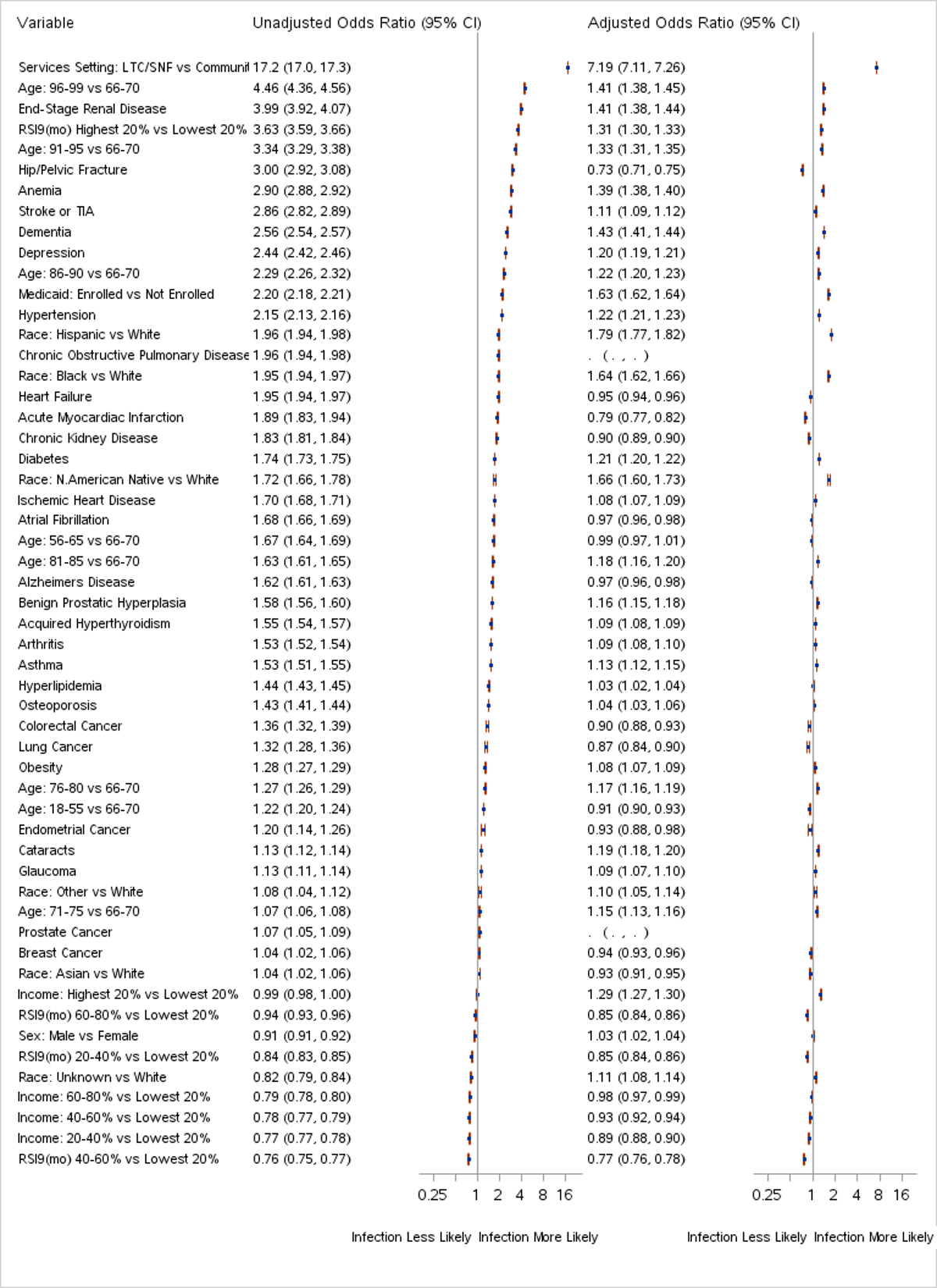

Supplement: S4 Fig — Confirmed Covid-19 cases were identified consistent with CMS guidance using ICD-10-CM codes for Covid-19 (B97.29 before April 1, 2020 and U07.1 thereafter) as a primary or secondary diagnosis between March 1, 2020 and September 30, 2020 [22]. Probable Covid-19 infection cases were identified using ICD-10-CM codes consistent with the CDC guidance (Z20.828) and WHO recommendations (U07.2) [23, 24]. Subjects were categorized as “LTC/SNF” if they received services in either a Long-Term Care (LTC) or Skilled Nursing Facility (SNF) in February 2020, otherwise they were categorized as receiving services in the “Community.” Predictors were assessed at baseline (February 29, 2020) and include quintiles of Risk Stratification Index (RSI), presence of chronic conditions, location of services (LTC/SNF vs Community), and demographic variables (i.e., age, sex, race, and quintiles of median household income imputed by zip code according to 2015 Census data.) Variables not remaining in the adjusted model are indicated by the presence of empty parenthesis under the adjusted odds ratio. Location of services, age, status of end-stage renal disease (ESRD) and RSI were the strongest (unadjusted) predictors of infection. Location of services and ESRD remained strong predictors following adjustment; however, risks associated with having chronic conditions were typically reduced when adjusted by the presence of other factors. (TIF) [file pone.0262264.s004.tif]

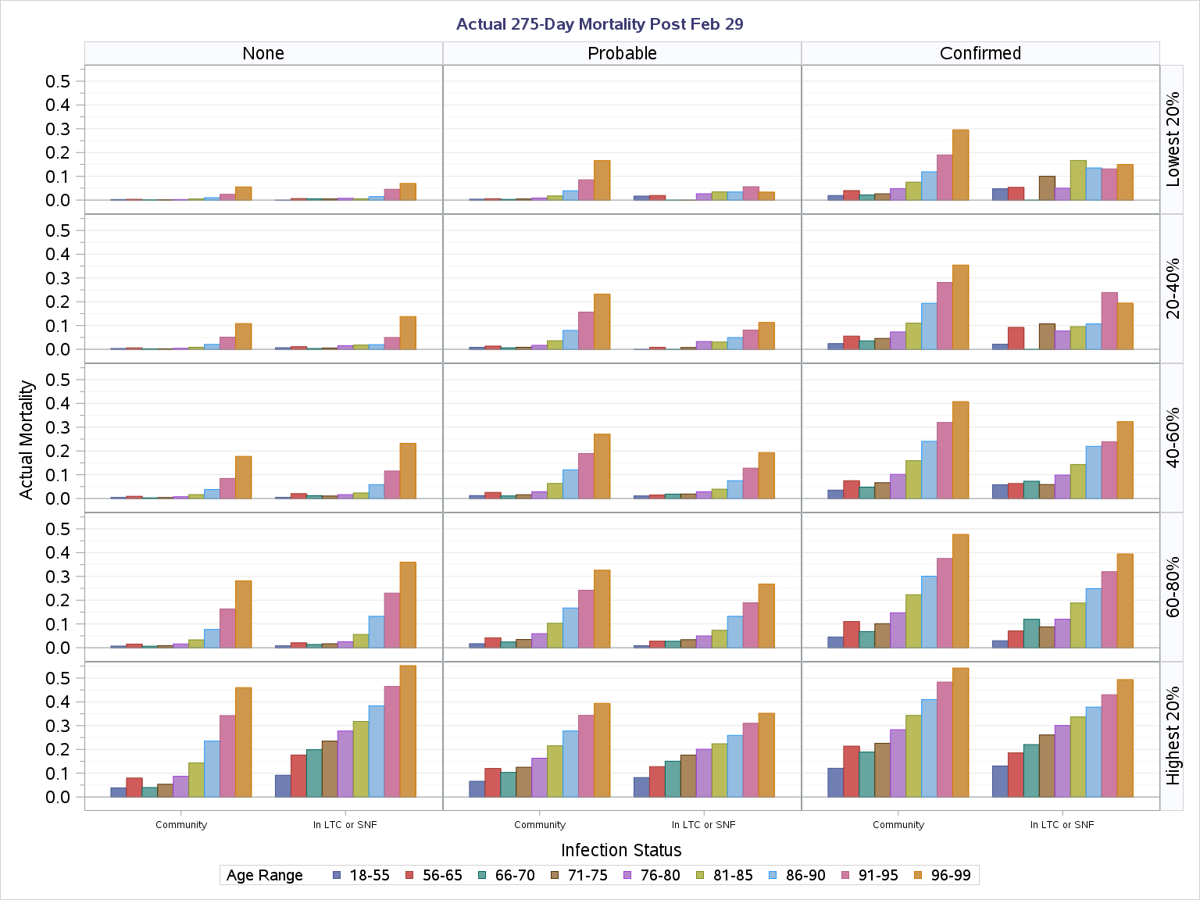

Supplement: S5 Fig — Rates of mortality within 9 months following baseline (February 29, 2020) in Medicare subpopulations categorized by age, location of services, infection status, and quintiles of the baseline risk of mortality assessed using the Risk Stratification Index (RSI). Confirmed Covid-19 cases were identified consistent with CMS guidance using ICD-10-CM codes for Covid-19 (B97.29 before April 1, 2020 and U07.1 thereafter) as a primary or secondary diagnosis between March 1, 2020 and September 30, 2020 [22]. Probable Covid-19 infection cases were identified using ICD-10-CM codes consistent with the CDC guidance (Z20.828) and WHO recommendations (U07.2) [23, 24]. Subjects were categorized as “LTC/SNF” if they received services in either a Long-Term Care (LTC) or Skilled Nursing Facility (SNF) in February 2020, otherwise they were categorized as receiving services in the “Community.” As expected, subjects in quintiles with higher baseline risk of mortality had higher rates of observed mortality. For subjects without a Covid diagnosis, mortality rates were lower in the community setting compared to those in the LTC/SNF; however, for subjects with confirmed or probable Covid infection, mortality rates were typically higher in the community setting than in the LTC/SNF. (TIF) [file pone.0262264.s005.tif]
