# Supplementary material for: Covid-19 and excess mortality in medicare beneficiaries
Source: PLoS One. 2022 Feb 2;17(2):e0262264. doi: 10.1371/journal.pone.0262264 (PMC8809573; doi:10.1371/journal.pone.0262264)
Supplement: S1 Table — Subjects were categorized as “LTC/SNF” if they received services in either a Long-Term Care (LTC) or Skilled Nursing Facility (SNF) in February 2020, otherwise they were categorized as receiving services in the “Community.” Confirmed Covid-19 cases were identified consistent with CMS guidance using ICD-10-CM codes for Covid-19 (B97.29 before April 1, 2020 and U07.1 thereafter) as a primary or secondary diagnosis between March 1, 2020 and September 30, 2020 [22]. Probable Covid-19 infection cases were identified using ICD-10-CM codes consistent with the CDC guidance (Z20.828) and WHO recommendations (U07.2) [23, 24]. The baseline risk of 9-month mortality defined by the Risk Stratification Index (RSI) calculated on February 29, 2020. Beneficiaries receiving a diagnosis of probable or confirmed Covid-19 were pairwise exactly matched 1:1 on Feb 29, 2020 with beneficiaries without a Covid-19 diagnosis based on sex, age (within 1-year), ethnicity, location of services in Feb 2020 (community or LTC/SNF), along with RSI as a propensity factor (within 0.1%). The tabulated results demonstrate the expected results where the percentage of unmatched cases is generally highest in the subpopulations within a category that are either the smallest (e.g., American Indians/Alaskan Native) or that generally do not use LTC/SNF services (e.g., the youngest population). (PDF) [file pone.0262264.s006.pdf]

S1 Table. Comparison of characteristics for Covid-19 subjects matched versus unmatched with controls in Community and LTC/SNF subgroups.

Characteristic	Study group, No. (%)											
	Community						LTC/SNF					
	COVID Confirmed			COVID Probable			COVID Confirmed			COVID Probable		
	Matched (n =470,622)	Unmatched (n=1,707)	% Un-matched	Matched (n =2,667,931)	Unmatched (n =8,571)	% Un-matched	Infected (n =186,671)	Unmatched (n=18,100)	% Un-matched	Infected (n =225,970)	Unmatched (n=15,132)	% Un-matched
**Age Group**												
18-55	35,622 (7.6)	850 (49.8)	2.3%	195,609 (7.3)	4,501 (52.5)	2.2%	4,591 (2.5)	2,574 (14.2)	35.9%	6,669 (3.0)	3,651 (24.1)	35.4%
56-65	40,029 (8.5)	202 (11.8)	0.5%	204,327 (7.7)	1,045 (12.2)	0.5%	14,739 (7.9)	2,685 (14.8)	15.4%	16,995 (7.5)	2,423 (16.0)	12.5%
66-70	98,466 (20.9)	99 (5.8)	0.1%	620,578 (23.3)	732 (8.5)	0.1%	17,537 (9.4)	2,434 (13.4)	12.2%	19,728 (8.7)	1,977 (13.1)	9.1%
71-75	98,994 (21.0)	95 (5.6)	0.1%	617,788 (23.2)	639 (7.5)	0.1%	21,884 (11.7)	2,562 (14.2)	10.5%	25,150 (11.1)	1,963 (13.0)	7.2%
76-80	74,957 (15.9)	106 (6.2)	0.1%	443,190 (16.6)	467 (5.4)	0.1%	26,201 (14.0)	2,661 (14.7)	9.2%	30,893 (13.7)	1,747 (11.5)	5.4%
81-85	55,589 (11.8)	98 (5.7)	0.2%	294,423 (11.0)	373 (4.4)	0.1%	31,053 (16.6)	2,134 (11.8)	6.4%	37,647 (16.7)	1,370 (9.1)	3.5%
86-90	38,729 (8.2)	95 (5.6)	0.2%	180,162 (6.8)	335 (3.9)	0.2%	33,772 (18.1)	1,699 (9.4)	4.8%	42,494 (18.8)	1,039 (6.9)	2.4%
91-95	22,229 (4.7)	102 (6.0)	0.5%	90,050 (3.4)	287 (3.3)	0.3%	27,166 (14.6)	958 (5.3)	3.4%	34,779 (15.4)	703 (4.6)	2.0%
96-99	6,007 (1.3)	60 (3.5)	1.0%	21,804 (0.8)	192 (2.2)	0.9%	9,728 (5.2)	393 (2.2)	3.9%	11,615 (5.1)	259 (1.7)	2.2%
**Sex**												
Female	255,638 (54.3)	821 (48.1)	0.3%	1,494,273 (56.0)	4,019 (46.9)	0.3%	119,501 (64.0)	9,063 (50.1)	7.0%	148,245 (65.6)	6,994 (46.2)	4.5%
Male	214,984 (45.7)	886 (51.9)	0.4%	1,173,658 (44.0)	4,552 (53.1)	0.4%	67,170 (36.0)	9,037 (49.9)	11.9%	77,725 (34.4)	8,138 (53.8)	9.5%
**Race/ Ethnicity**												

American Indian/ Alaskan Native	4,115 (0.9)	266 (15.6)	6.1%	14,858 (0.6)	1,249 (14.6)	7.8%	263 (0.1)	674 (3.7)	71.9%	389 (0.2)	740 (4.9)	65.5%
Asian/ Pacific Islander	11,587 (2.5)	89 (5.2)	0.8%	52,726 (2.0)	842 (9.8)	1.6%	2,868 (1.5)	1,725 (9.5)	37.6%	2,339 (1.0)	3,146 (20.8)	57.4%
Black	64,355 (13.7)	254 (14.9)	0.4%	235,296 (8.8)	1,689 (19.7)	0.7%	25,511 (13.7)	7,364 (40.7)	22.4%	21,847 (9.7)	4,894 (32.3)	18.3%
Hispanic	50,242 (10.7)	233 (13.6)	0.5%	138,907 (5.2)	1,059 (12.4)	0.8%	10,616 (5.7)	852 (4.7)	7.4%	8,160 (3.6)	824 (5.4)	9.2%
Non-Hispanic White	329,199 (69.9)	217 (12.7)	0.1%	2,156,397 (80.8)	1,042 (12.2)	0.0%	146,674 (78.6)	2,222 (12.3)	1.5%	192,426 (85.2)	1,635 (10.8)	0.8%
Other	3,627 (0.8)	323 (18.9)	8.2%	18,005 (0.7)	1,311 (15.3)	6.8%	452 (0.2)	4,772 (26.4)	91.3%	468 (0.2)	3,207 (21.2)	87.3%
Unknown	7,497 (1.6)	325 (19.0)	4.2%	51,742 (1.9)	1,379 (16.1)	2.6%	287 (0.2)	491 (2.7)	63.1%	341 (0.2)	686 (4.5)	66.8%
**Low Income/ Disabled Status**												
Low Income or Disabled	188,443 (40.0)	249 (14.6)	0.1%	839,214 (31.5)	1,428 (16.7)	0.2%	123,380 (66.1)	2,123 (11.7)	1.7%	123,488 (54.6)	2,880 (19.0)	2.3%
Not Low Income or Disabled	282,179 (60.0)	1,458 (85.4)	0.5%	1,828,717 (68.5)	7,143 (83.3)	0.4%	63,291 (33.9)	15,977 (88.3)	20.2%	102,482 (45.4)	12,252 (81.0)	10.7%
**RSI (9mo)**												
MEAN (SD)	0.048 (.085)	0.13 (0.15)	NA	0.038 (.076)	0.15 (0.16)	NA	0.212 (.136)	0.15 (0.13)	NA	0.205 (.140)	0.13 (0.14)	NA
**Mortality (through Nov 30)**												
Died	69,053 (14.7)	435 (25.5)	0.6%	165,491 (6.2)	1,309 (15.3)	0.8%	57,312 (30.7)	4,600 (25.7)	7.4%	45,745 (20.2)	2,057 (13.6)	4.3%
Survived	401,569 (85.3)	1,272 (74.5)	0.3%	2,502,440 (93.8)	7,262 (84.7)	0.3%	129,359 (69.3)	13,400 (74.3)	9.4%	180,225 (79.8)	13,075 (86.4)	6.8%
